# Volleying plasma membrane proteins from birth to death: Role of J-domain proteins

**DOI:** 10.3389/fmolb.2022.1072242

**Published:** 2022-12-15

**Authors:** Preeti Sagarika, Kirpa Yadav, Chandan Sahi

**Affiliations:** Department of Biological Sciences, IISER Bhopal, Bhopal, India

**Keywords:** J-domain proteins (JDPs), Hsp70, plasma membrane proteins, protein quality control (PQC), secretory pathway

## Abstract

The function, stability, and turnover of plasma membrane (PM) proteins are crucial for cellular homeostasis. Compared to soluble proteins, quality control of plasma membrane proteins is extremely challenging. Failure to meet the high quality control standards is detrimental to cellular and organismal health. J-domain proteins (JDPs) are among the most diverse group of chaperones that collaborate with other chaperones and protein degradation machinery to oversee cellular protein quality control (PQC). Although fragmented, the available literature from different models, including yeast, mammals, and plants, suggests that JDPs assist PM proteins with their synthesis, folding, and trafficking to their destination as well as their degradation, either through endocytic or proteasomal degradation pathways. Moreover, some JDPs interact directly with the membrane to regulate the stability and/or functionality of proteins at the PM. The deconvoluted picture emerging is that PM proteins are relayed from one JDP to another throughout their life cycle, further underscoring the versatility of the Hsp70:JDP machinery in the cell.

## Introduction

The plasma membrane (PM) is a multi-protein collage where proteins are frequently clustered and embedded in the lipid bilayer. PM proteins broadly consist of integral and peripheral proteins. Together, they perform numerous cellular functions, such as cell anchoring and cell-cell interactions, regulating cellular nutrient uptake and ion homeostasis, as well as integrating cellular physiology with the environment. Moreover, they are critical for the structural integrity of the PM (Alberts B et al., 2002; Bernardino de la Serna et al., 2016). Therefore, preserving the functionality and the heterogeneity of these cell surface proteins is crucial. To accomplish this, cells have an intricate internal membrane system that mediates their insertion and removal from the plasma membrane (Okiyoneda et al., 2011; MacGurn, 2014; Sardana and Emr, 2021).

PM proteins are extremely hydrophobic and are embedded, partially or completely in the lipid bilayer. Hence, the biogenesis, trafficking, stability, and degradation of PM proteins are far more challenging than soluble cytosolic proteins. Cells are extremely sensitive to conformationally compromised PM proteins, and often this leads to loss of cell integrity or death (Zhao et al., 2013; Juarez-Navarro et al., 2020). To circumvent this, cells employ efficient, multi-layered cellular protein quality control (PQC) machineries which act as stringent checkpoints to tightly regulate the synthesis, folding, and degradation of PM proteins (Reviewed in (Apaja et al., 2010; Okiyoneda et al., 2011; Babst, 2014; MacGurn, 2014; Sardana and Emr, 2021; Vasconcelos-Cardoso et al., 2022). At each of these steps, PM proteins must pass the “fit to move forward” test. In case they do not meet the quality control (QC) standards, they are appropriately handled by one or the other component(s) of the cellular PQC machinery (Anelli and Sitia, 2008; Okiyoneda et al., 2011; Sun and Brodsky, 2019; Phillips et al., 2020; Schwabl and Teis, 2022). At the PM, too, these proteins are constantly at risk of misfolding or aggregation. Furthermore, their stability and/or abundance at the PM is also very tightly regulated; for example, for many nutrient transporters, mild temperature shifts or availability of substrate promotes their endocytosis and degradation (Apaja et al., 2010; Ghaddar et al., 2014; Rodriguez-Furlan et al., 2019).

Molecular chaperones, mainly the Hsp70:J-domain protein (JDP) machinery (see Box 1), play a major role in regulating cellular proteostasis (Rosenzweig et al., 2019; Liu et al., 2020; Zhang et al., 2022). In the last two decades, the essential requirement of chaperones for various PQC pathways has been extensively highlighted. Paradoxically, even though PM proteins pose greater challenges to PQC machinery compared to cytosolic proteins, the involvement of chaperones in the QC of PM proteins is less understood. Nevertheless, different Hsp70s and JDPs have been implicated in assisting PM proteins in their biogenesis, maturation, trafficking, and degradation in different organisms. Moreover, the ability of selected Hsp70s and JDPs to directly associate with lipids and membranes sheds light on their possible role in the PM as well (Caplan et al., 1992; Kanazawa et al., 1997; Beilharz et al., 2003; Kalli et al., 2013; Radons, 2016; Sopha et al., 2017; Sjögren et al., 2018; Dobriyal et al., 2020; De Maio and Hightower, 2021; Zhang et al., 2021) (Table 1). The importance of different chaperones and other PQC factors in diseases associated with PM proteins is discussed elsewhere (Juarez-Navarro et al., 2020). In this review, we collate data available from multiple organisms, including yeast, plants, and mammalian models, and come up with a unifying model to show that, like cytosolic proteins, the versatile Hsp70:JDP systems are guardians of PM proteins as well, throughout their odyssey in the cell (Table 2).

**TABLE 1:** List of Lipid interacting cytosolic JDPs.

JDPs	Known lipid interaction domain	References
Yeast		
Hlj1	Tail-anchored	(Beilharz et al., 2003; Youker et al., 2004)
Ydj1	Anchored by Farnesylation at C-terminal CAAX domain	(Caplan et al., 1992)
Sec63, ERj5	Transmembrane domain	(Feldheim et al., 1992)
Caj1	Charged residues	(Herianto et al., 2021; Zhang et al., 2021)
Human		
DNAJB 12,14	ER transmembrane proteins	(Sopha et al., 2017)
DNAJA1, A2	Anchored by Farnesylation at C-terminal CAAX domain	(Kanazawa et al., 1997; Terada and Mori, 2000)
ERdj1,2	Transmembrane domain	(Dudek et al., 2005, p. 1; Skowronek et al., 1999)
Auxilin 1, 2	PTEN-like domain	(Kalli et al., 2013; Lee et al., 2006)
Arabidopsis		
AtDJA1, A2 (J2, J3)	Anchored by Farnesylation at C-terminal CAAX domain	(Barghetti et al., 2017; Sjögren et al., 2018)
AtERdj1,2,7	Transmembrane domain	(Ohta and Takaiwa, 2014)

**TABLE 2:** List of JDPs in mediating the PQC of PM proteins in yeast, human, and Arabidopsis.

Steps	Pathways	Hsp70	JDP	Organism	References
Biosynthesis/ER targeting	Co-translational ER targeting	Ssb1/2	Zuo1	Yeast	(Pfund et al., 1998)
		Hsp70L1	DNAJC2	Human	(Hundley et al., 2005)
		−	AtDjC1 (AT3G11450)^#^, AtDjC2 (AT5G06110)^#^	Arabidopsis	(Verma et al., 2017)
	Chaperone-mediated post-translational Targeting	Ssa1^*^	Ydj1*	Yeast	(Caplan et al., 1992)
		Hsc70	Unknown JDP	Human	(Abell et al., 2007)
		atHsp70-1^#^	−	Arabidopsis	(Schweiger and Schwenkert, 2013)
	Post-translational targeting by GET/TRC40 pathway	Ssa1	Apj1, Jjj3, Sis1 and Ydj1	Yeast	(Ast et al., 2013; Cho et al., 2021)
		Hsc70	Hsp40	Human	(Abell et al., 2007; Rabu et al., 2008)
		−	−	Arabidopsis	−
ER import	Import and lipid insertion in ER	Kar2	Sec63	Yeast	(Jung et al., 2019; Sadler et al., 1989)
		BiP	ERdj1, ERdj2	Human	(Dudek et al., 2005; Skowronek et al., 1999)
		AtBiP1*, AtBiP2*, AtBiP3*	At1G79940/AtERdj2A^#^, At4G21180/AtERdj2B^#^	Arabidopsis	(Maruyama et al., 2014; Ohta and Takaiwa, 2014)
Protein quality control in ER	Quality control by ER luminal factors	−	−	Yeast	(Mehnert et al., 2015)
		BiP	Erdj3*, ERdj4, ERdj5, ERdj6^#^	Human	(Dong et al., 2008; Otero et al., 2010)
		AtBiP1	AtERdj3A (AT3G08970)^#^, AtERdj3B (AT3G62600), AtP58IPK (AT5G03160)^#^	Arabidopsis	(Li et al., 2009; Nekrasov et al., 2009; Ohta and Takaiwa, 2014)
	Quality control by cytosolic factors	Ssa1-4	Ydj1, Hlj1	Yeast	(Youker et al., 2004)
		Hsp70(HSPA1A), Hsc70 (HSPA8)	DNAJA1, DNAJA2, DNAJA4, DNAJB1, DNAJB12, DNADB14	Human	(Farinha et al., 2002; Grove et al., 2011; Meacham et al., 1999; Sopha et al., 2012; Walker et al., 2010)
		−	−	Arabidopsis	
Protein quality control at PM		NA	Caj1	Yeast	(Dobriyal et al., 2020)
		Hsc70, Hsp70	DNAJA1, DNAJB2, DNAJA2	Human	(Bagdany et al., 2017; Okiyoneda et al., 2010)
		−	−	Arabidopsis	
Clathrin-mediated endocytosis		Ssa1	Swa2	Yeast	(Xiao et al., 2006)
		Hsc70	DNAJC6, GAK	Human	(Greener et al., 2000)
		AtHsc70	AUXILIN-LIKE1 (At4g12780), AUXILIN-LIKE2 (At4g12770), AUXILIN-LIKE3 (At1g21660)^#^, AUXILIN-LIKE4 (At4g36520)^#^, AUXILIN-LIKE5 (At1g75310)^#^, JAC1/AUXILIN-LIKE6 (At1g75100)^#^, AUXILIN-LIKE7 (At1g30280)^#^	Arabidopsis	(Adamowski et al., 2018; Lam et al., 2001; Verma et al., 2017)

*Predicated based on known function and literature on other substrates. ^#^Predicted based on sequence similarity/domain organization.

## PM proteins: The PQC machinery’s nightmare

**FIGURE 1:**
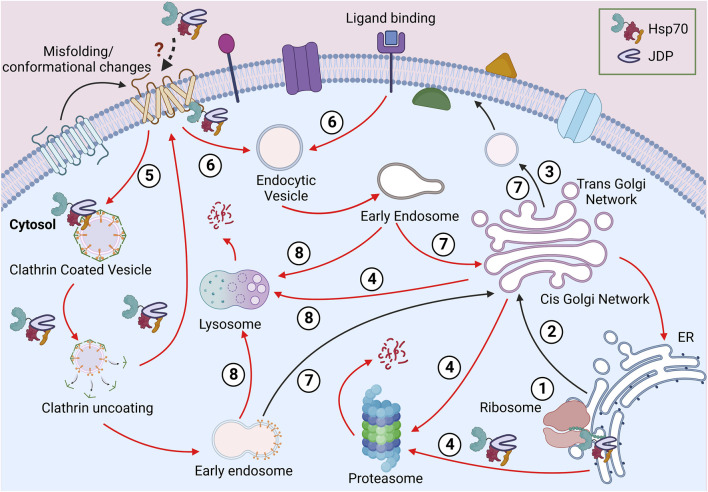
Journey of a PM protein. Soon after the ribosomal biosynthesis, the newly synthesized PM protein translocates to the ER (1). The well-folded proteins are packed in vesicles and transported to the PM *via* the Golgi apparatus (2-3). The misfolded proteins from the endomembrane system can be directed for proteasomal or lysosomal degradation (4). Due to misfolding or stimuli-based conformational change at the PM, proteins can be packaged in clathrin-independent (5) or clathrin-dependent (6) vesicles. These proteins can be recycled to PM (7) or targeted for lysosomal degradation (8). Different steps requiring Hsp70:JDPs are shown in the diagram.

Maintaining the proper QC of PM proteins is one of the major challenges faced by cellular PQC machinery. Like other membrane proteins, PM proteins are folded into different conformations and topologies governed by their primary structures. Many membrane proteins are known to have dual or multiple topologies, which adds to the complexity of the plasma membrane PQC (PMPQC) (von Heijne, 2006; Woodall et al., 2015). There are different types of membrane proteins, including transmembrane proteins, which are amphipathic. They harbor one or more hydrophobic segments, spanning the lipid bilayer, and hydrophilic domains, that stay exposed to the cytosol and extracellular space (Deol et al., 2004). Several other PM proteins are anchored to the lipid bilayer either through a hydrophobic segment a covalent lipid (fatty acid chain or a prenyl group), or a sugar modification. PM proteins have a complex life cycle (shown in Figure 1). They follow the secretory pathway through the cellular endomembrane system to reach their destination. *En route*, they pass through different sub-cellular environments with varying pH, ionic concentrations, and redox states (Anelli and Sitia, 2008; Delic et al., 2013). PM proteins are synthesized on the ribosomes and are co- or post-translationally targeted to the ER. Then they are incorporated into the ER membrane to undergo folding and maturation, which involves the participation of both ER and cytosolic factors (Sun and Brodsky, 2019; Phillips et al., 2020). ER provides an oxidative environment for the disulfide bond formation and proper folding. ER-residing enzymes are essential for adding GPI anchors to the anchored PM proteins. The assembly and maturation of proteins are followed by their packaging into vesicles and targeting to the Golgi apparatus for further post-translational modifications (PTMs) and sorting to PM. These organelles have different pH and provide a protective environment to the proteins until they reach the cell surface. The Golgi apparatus and vesicles are slightly acidic compared to the cytosol and ER (pH 7-7.5). Any change in the pH (pH 6.2-6.8 at *cis*-Golgi to pH 6.0-6.3 at the *trans*-Golgi network) or ion (H^+^, Ca^2+^, Mg^2+^, Mn^2+^) concentrations can lead to perturbed Golgi functions (Shen et al., 2013; Kellokumpu, 2019).

Being the cellular periphery, at the PM, proteins are often exposed to various physical, mechanical, or chemical stress, risking their native structure and function. Additionally, the lipid composition of the PM too plays a vital role in maintaining the local structure, dynamics, stability, and functionality of PM proteins (Lenaz, 1987; Lee, 2004; Bukiya and Dopico, 2019; Fratti, 2021). For example, the localized lipids interact with the G-protein coupled receptors (GPCR) and affect conformation, stability and signaling at the membrane (Mahmood et al., 2013; Desai and Miller, 2018). Similarly, polyunsaturated fatty acids (PUFA) interact with several voltage-gated ion channels to regulate their activity (Elinder and Liin, 2017). Apart from the direct interactions, localized lipid rafts also form membrane compartments and, in turn, regulate the function, stability, sorting, maturation, and turnover of proteins (Casares et al., 2019; Lujan and Campelo, 2021). Even the final stages of PM proteins’ life cycle are not simple (Figure 1). The removal of proteins from PM follows the endocytic route, where the proteins are packaged into vesicles and targeted to early endosomes (Zhao et al., 2013; Ghaddar et al., 2014; Claus et al., 2018). These proteins are further targeted to lysosome/vacuole for degradation. However, endocytosis does not ensure degradation as, in some cases, PM proteins are modified to their native structure and recycled back to the PM (Claus et al., 2018; Rodriguez-Furlan et al., 2019; Sardana and Emr, 2021).

Hence the complex life cycle of PM proteins counts on the proper functioning of multiple PQC machineries that work in parallel in different subcellular compartments. It is very likely that these heterogeneous groups of proteins require dedicated cellular factors during the complex journey inside the cell.

## Targeting to ER

**FIGURE 2:**
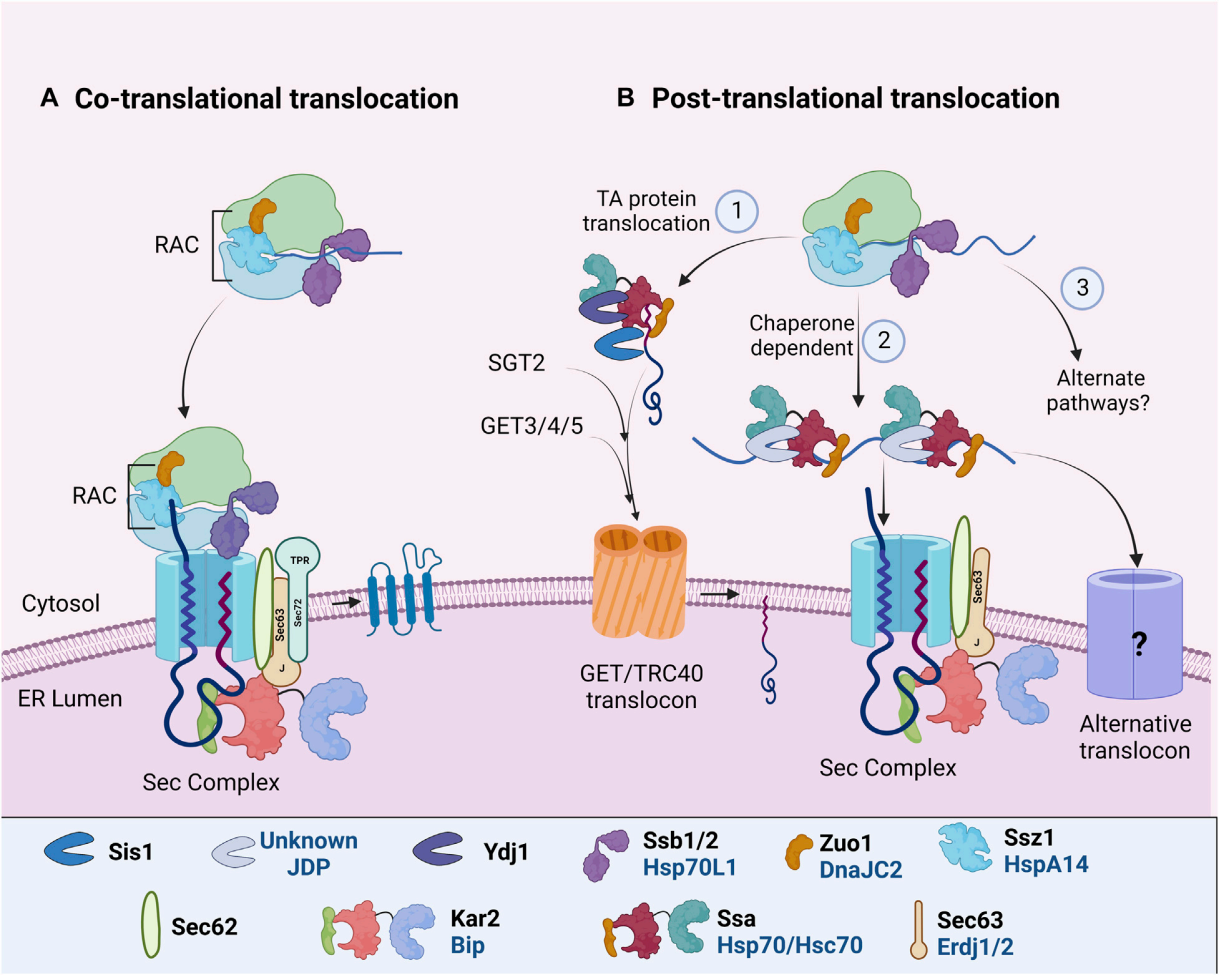
ER targeting and import of PM proteins. In different organisms, specific Hsp70: JDPs mediate the ER targeting of PM proteins by **(A)** CTT or **(B)** PTT. During both CTT and PTT, ribosome-associated Hsp70:JDPs^Y,H^ interact and protect the nascent polypeptides (Otto et al., 2005; Döring et al., 2017; Chen et al., 2022). PTT occurs through multiple pathways (1) GET/TRC40 mdiated^Y,^ (2) Chaperone mediated^H^ or (3) alternate pathways^N^ (Rabu et al., 2009; Casson et al., 2017; Cho et al., 2021). Further, the ER-localized Hsp70:JDPs^Y, H, A^ perform the protein import and lateral insertion of PM proteins (Ohta and Takaiwa, 2014; Pobre et al., 2019; Itskanov et al., 2021, p. 63). ^Y^ Literature available in Yeast; ^H^ Humans; ^A^ Arabidopsis. ^N^ No literature available. Names of Hsp70 and JDPs are denoted in different colors; Black for Yeast; Blue for Human.

Almost all PM proteins follow the secretory pathway to reach their destination. This begins with the translocation of PM proteins to the endoplasmic reticulum (ER); either co-translationally, where protein synthesis is coupled with protein import into the ER or post-translationally, where protein translocation takes place after the complete polypeptide is synthesized (reviewed by (Rapoport, 2007; Cross et al., 2009; Hegde and Keenan, 2011; Zimmermann et al., 2011; Aviram and Schuldiner, 2017; Linxweiler et al., 2017). The mode of ER import depends on the amino acid composition of the polypeptide. Different Hsp70:JDP machineries assist these pathways to stabilize the translation elongation, prevent misfolding, aggregation, or facilitate interaction with the ER translocon complex (Ast et al., 2013; Aviram and Schuldiner, 2017; Chen et al., 2022) (Figure 2). During protein synthesis, the Ssb1/2 (yeast)/Hsp70L1 (mammals), together with Ribosome Associated Complex (RAC), functions to regulate the folding and targeting of nascent polypeptides to the ER. RAC is a heterodimer composed of Hsp70, Ssz1 (HSPA14 in mammals), and JDP, Zuo1 (DNAJC2 in mammals) (Hundley et al., 2005; Otto et al., 2005). Upon nascent chain elongation, Ssz1 and Zuo1 undergo a conformational change, thus making the J-domain of Zuo1 accessible to binding with another Hsp70, Ssb1/2 (Chen et al., 2022). Ssb interacts with a large number of ribosome-associated nascent chains as well as the cytosolic factor, signal recognition particle (SRP), to facilitate efficient translation and ER targeting of proteins (Pfund et al., 1998; Willmund et al., 2013; Döring et al., 2017; Shiber et al., 2018). Together, Ssb and Zuo1 help in co-translational protein folding and their targeting to the ER (Kramer et al., 2019; Chen et al., 2022). Although deletion of Ssb leads to widespread misfolding and aggregation of newly synthesized polypeptides (Koplin et al., 2010), its requirement in the biogenesis or maturation of membrane proteins is unknown. While Ssb preferentially interacts with cytosolic and nuclear proteins and less with membrane proteins (Willmund et al., 2013), the sensitivity of yeast cells lacking Ssb1/2 or the partner JDP, Zuo1, to aminoglycosides is linked to an increase in cation influx as a consequence of altered maturation of membrane proteins (Kim and Craig, 2005). It suggests that Ssb1/2, along with Zuo1 may regulate the biogenesis of PM proteins as well (Peisker et al., 2010).

### Co-translational translocation

Many of the transmembrane PM proteins undergo co-translational translocation (Figure 2A). Co-translationally translocated proteins usually harbor a cleavable hydrophobic N-terminal signal sequence (SS) or a non-cleavable transmembrane (TM) domain. As soon as the polypeptide emerges from the ribosome, SRP recognizes and binds the ribosome nascent chain complex (RNC) and targets it to the Sec61 complex at the ER membrane (Pool et al., 2002; Halic et al., 2004; Linxweiler et al., 2017). As the RNC interacts with the translocon, the peptide is inserted co-translationally into the ER until the synthesis of a hydrophobic TM segment (i.e., stop transfer sequence) terminates nascent chain translocation. TM segments move laterally out of the translocon as they integrate into the lipid bilayer (Figure 2). In recent years, an alternate ER targeting mechanism involving Snd2p and Snd3p (SRP-iNDependent targeting) has been reported to target substrates to the Sec61 complex. It can partially compensate for the loss of both SRP-mediated and post-translationally targeted substrates. However, whether it operates co-translationally or post-translationally is yet to be understood (Aviram et al., 2016; Haßdenteufel et al., 2017; Lei et al., 2020). The co-translational translocation pathways prevent the exposure of nascent polypeptides to the cytosol. Although ribosome-associated Hsp70:JDPs regulate the process, there is no report of the involvement of additional cytosolic chaperones.

### Post-translational translocation

In post-translational translocation (PTT), fully translated polypeptides are targeted to the ER translocon. Proteins remain unfolded for their translocation through ER. Hence cytosolic factors, specifically Hsp70s and JDPs, associate with the polypeptide to prevent intermolecular interaction and aggregation, as well as facilitate interaction with the translocon complex (Figure 2B). A substantial number of proteins are translocated post-translationally into the ER in yeast as well as in complex eukaryotes (Ast et al., 2013; Ast and Schuldiner, 2013; Aviram and Schuldiner, 2014). For PTT of membrane proteins, specifically tail-anchored (TA) proteins, at least two distinct yet overlapping pathways have been reported. First is the GET/TRC40 pathway, targeting TA proteins to the GET/TRC40 receptor complex at ER, and second, the chaperone-mediated pathway, targeting some secretory and TA proteins to ER Sec translocon complex.

#### GET/TRC40 pathway

Tail-anchored (TA) proteins harbor a single transmembrane domain at their C-terminus (Ast et al., 2013; Casson et al., 2017). A major pathway for TA protein targeting is by the guided entry of tail-anchored proteins (GET) or transmembrane recognition complex of 40 kDa (TRC40/ASNA1) pathway in yeast and mammals respectively (Favaloro et al., 2010, 2008; Schuldiner et al., 2008). In Arabidopsis, multiple GET pathway components have been identified and predicted to be involved in the translocation of TA proteins (Srivastava et al., 2017; Xing et al., 2017; Anderson et al., 2021; Asseck et al., 2021), but our understanding of the Hsp70:JDPs in this process is far from complete. A pre-targeting complex composed of cytosolic chaperone Sgt2 (yeast)/SGTA (mammals) and the Get4-Get5 heterodimer (yeast)/TRC35-UBL4A-BAG6 complex (mammals) recognizes the nascent TA polypeptide that further interacts with the ATP-bound Get3 (yeast)/TRC40 (mammals). This transfers the TA protein to Get3/TRC40 translocon complex for ER insertion (Jonikas et al., 2009; Mariappan et al., 2011; Wang et al., 2011).

In yeast and mammals, JDP (Ydj1) and Hsp/c70 physically interact with Sgt2/SGTA (Wu et al., 2001; Liou et al., 2007; Chartron et al., 2011). Recent studies in yeast have identified Hsp70:JDP machinery as the upstream component of this pathway (Cho et al., 2021) (Figure 2B). Hsp70, Ssa1, captures the TA polypeptide, which is further transferred to the chaperone component, Sgt2. While Ydj1 helps Ssa1 to capture nascent TA polypeptides and prevent their aggregation, both Ydj1 and Sis1 (another cytosolic JDP) enhance the transfer of TA polypeptides to Sgt2. The initial client capture by Ydj1 requires both the functional J-domain as well as the client binding domain, while only J-domain is sufficient for TA polypeptide’s transfer to Sgt2. The study clearly suggests that JDPs not only capture substrates but also induce conformational changes to facilitate their transfer to downstream factors and subsequently to the ER. Ydj1 needs special mention here as it is tethered to the ER membrane through c-terminal farnesyl modification, which is important for targeting proteins to the ER (Caplan et al., 1992; Ast et al., 2013). Additionally, yeast cytosolic JDPs, Apj1 and Jjj3, are also implicated in targeting GPI-anchored protein Gas1 to ER (Ast et al., 2013) through unknown mechanisms.

#### Chaperone-mediated PTT

In humans, the insertion of TA proteins seems to be more complex and substrate-specific. The TRC40/GET mediated pathway, although specific, is not essential for targeting all TA proteins (Coy-Vergara et al., 2019). Alternative pathways involving Hsc70:JDPs, signal recognition particle (SRP), or Sndp (SRP-iNDependent targeting), execute client targeting to the ER (Abell et al., 2007; Rabu et al., 2009, 2008; Aviram et al., 2016; Casson et al., 2017; Farkas and Bohnsack, 2021) (Figure 2B). Hsc70 functions with JDPs to promote the ATP-dependent membrane targeting of the TA protein, Sec61β, and other TA precursors *in vitro* (Abell et al., 2007). However, inhibition of Hsc70 affected only a subset of TA proteins’ integration in Hela cells ER, suggesting functional redundancy with other Hsp70s or other parallelly operating pathways (Rabu et al., 2008).

The Hsp70:JDP does not only interact with clients but also with the ER translocon. Studies reveal that yeast Ssa1, through its c-term EEVD motif, and Ssb1, through its nucleotide-binding domain, interact with the tetratricopeptide repeat (TPR) domain of Sec72 translocon (Tripathi et al., 2017). Similarly, in Arabidopsis, constitutively expressed AtHsp70-1 interacts with the ER translocation machinery component AtTPR7 (Schweiger et al., 2012; Schweiger and Schwenkert, 2013). Although metazoans do not have a Sec72 ortholog, one of the ER membrane JDPs, Erdj1, regulates translation and protein import into the ER (Blau et al., 2005). However, the precise mechanism of how exactly the JDPs regulate PTT in humans, is still unknown. Several questions remain unanswered; for example, 1) at which step Hsc70:JDPs act on the proteins, 2) what drives its substrate specificity, 3) whether it is in parallel or in the same pathways as TRC40, and 4) how it interacts with the ER translocon in the absence of Sec72.

## Import into the ER

ER is the hub of protein folding and maturation, and it also acts as the first PQC checkpoint for PM proteins. Unsurprisingly, the number and complexity of the Hsp70:JDP network in the ER is only next to the cytosol. Moreover, the ER-localized Hsp70:JDP complex is conserved across yeast, mammals, and plants. Budding yeast has a single Hsp70 (Kar2) and four JDPs, Scj1, Jem1, Erj5 and Sec63 (Nishikawa et al., 2005), and mammals have 7 JDPs Erdj1-7 working with Hsp70, BiP. Out of all these, yeast Sec63 and mammalian ERdj1/2 aid in the protein import across ER membrane, while the other JDPs are proposed to maintain the ER PQC (Kampinga and Craig, 2010; Otero et al., 2010; Melnyk et al., 2015). The JDP, Sec63/Erdj2, is a transmembrane protein and is an integral part of the conserved Sec61 translocon complex. It partners with Hsp70, Kar2/BiP, through its luminal J-domain to facilitate ATP-dependent unidirectional import of proteins (Corsi and Schekman, 1997; Misselwitz et al., 1999, 1999; Melnyk et al., 2015; Pobre et al., 2019; Daverkausen-Fischer and Pröls, 2021) (Figure 2). Sec63 lacking the N-term residues (Sec63_ΔN39) is severely impaired in the sorting of membrane proteins (Jung et al., 2019). Along with import, Sec63 also helps in the lateral gate opening of the Sec61 channel, thus helping in the lateral insertion of proteins into the ER membrane (Itskanov et al., 2021). Hence, it is an essential component of the ER translocon pathway. Sec63 deletion in yeast is lethal, and in humans, mutations in Sec63/Erdj2 are linked to autosomal dominant polycystic liver disease (PCLD) (Sadler et al., 1989; Davila et al., 2004; Janssen et al., 2012).

Apart from Erdj2, Erdj1 is also membrane-localized with an N-terminal luminal J-domain and C-terminal cytosolic domain, wherein the cytosolic domain binds ribosomes and inhibits translation (Dudek et al., 2005). Interestingly, this regulatory inhibition is released when Erdj1 binds to BiP in the ER lumen. It has been hypothesized that Erdj1 regulates translation in response to ER stress through its interaction with BiP (Blau et al., 2005; Benedix et al., 2010). However, under unstressed conditions, it may have protein import functions, possibly redundant with Erdj2.

In Arabidopsis there are six ER-localized JDPs (AtP58IPK, AtERdj2A, AtERdj2B, AtERdj3A, AtERdj3B, and AtERdj7). The ER luminal Hsp70, AtBiP, is present as three isoforms in Arabidopsis, AtBiP1, AtBiP2, and AtBiP3 (Maruyama et al., 2010). They probably interact with the J-domain of AtERdj2A/B (Sec63 orthologs) to perform the energy-dependent protein import in the ER (Yamamoto et al., 2008; Ohta and Takaiwa, 2014). However, it is important to further understand the redundancy and/or specificities of the Hsp70:JDP network in Arabidopsis. Although both AtERdj2A and AtERdj2B mediate protein import, they may interact with different substrates or different AtBiP paralogs, leading to diverse consequences. More detailed studies will help us elucidate their substrate specificity and novel mechanisms of protein import and their insertion in the ER membrane in plants. Non-etheless, the increased number of JDPs in ER from yeast to humans, and plants posits that besides functional redundancy, and making the system more robust, JDPs may also have unique functions as well (Blau et al., 2005; Dudek et al., 2005; Yamamoto et al., 2008; Benedix et al., 2010).

## Protein quality control in the ER

Once proteins reach the ER, they undergo folding and maturation. They require a variety of ER chaperones and co-chaperones for the same. Consistent with their structural heterogeneity, PM proteins undergo several maturation steps that require the formation of cis-trans prolyl isomers, disulfide bonds in the oxidizing ER environment, and the adoption of complex transmembrane topologies. Also, the addition, formation, and/or modification of sugar or lipid moieties is crucial for the maturation of several PM proteins (Braakman and Bulleid, 2011). The ER quality control (ERQC) machinery provides a suitable environment for their maturation and regulates the stoichiometry/concentration of subunits to favor correct assembly. Apart from the quality of proteins, ERQC also regulates the quantity of proteins by regulating their secretion according to the physiological requirements of the cell. Once the protein is folded and assembled, it is packaged into vesicles and transported to the Golgi apparatus.

### Role of luminal JDPs

In the ER lumen, yeast Kar2, Jem1, and Scj1; Human BiP and ERdj3-6, and Arabidopsis AtBiP1-3, AtERdjs maintain the QC of the luminal domains of trans-membrane proteins and soluble luminal proteins. They not only interact with protein substrates but also regulate the ER stress signaling pathways. For e.g., ERdj4 interacts with the key UPR (Unfolded Protein Response) (Box 2) signal activator, Ire1, and recruits BiP to suppress the UPR signaling in Chinese hamster ovary (CHO) cells (Amin-Wetzel et al., 2017). Similarly, in yeast and Arabidopsis, Kar2/AtBiP1-3 regulates UPR induction (Noh et al., 2003; Moreno et al., 2012). Although none of the Arabidopsis JDPs are yet identified to directly regulate Ire1, AtERdj3B interacts with SDF2, another crucial UPR regulator (Nekrasov et al., 2009). AtBiP1-3 and AtERdjs regulate the folding and maturation of a variety of PM proteins, specifically pattern-recognition receptors (PRRs), thus regulating their surface expression (Li et al., 2009; Saijo, 2010; Gupta and Tuteja, 2011; Park and Seo, 2015). Moreover, AtERdj3 interacts with AtBiP1-3 to regulate the biogenesis of surface receptor EFR (PRRs for EF-Tu), thereby playing an important role in regulating plant immune response (Nekrasov et al., 2009).

The role of luminal Hsp70:JDPs are not restricted to the ER. Human ERdj3, 4, and 6 show dual topology, which may also tether them to the ER membrane to perform different functions (Araki and Nagata, 2011; Daverkausen-Fischer and Pröls, 2021). ERdj3 is predicted to have two transmembrane domains and it interacts with clients as well as the Sec translocon. Interestingly, under UPR stress, Erdj3 is reported to be overexpressed and exported to the extracellular space to maintain proteostasis (Genereux et al., 2015). Also, under stress or apoptosis, BiP and the lectin calreticulin are trafficked to the PM (Zhang et al., 2010; Genereux et al., 2015), further underscoring the versatility of ER luminal Hsp70s and JDPs.

### Role of cytosolic JDPs

**FIGURE 3:**
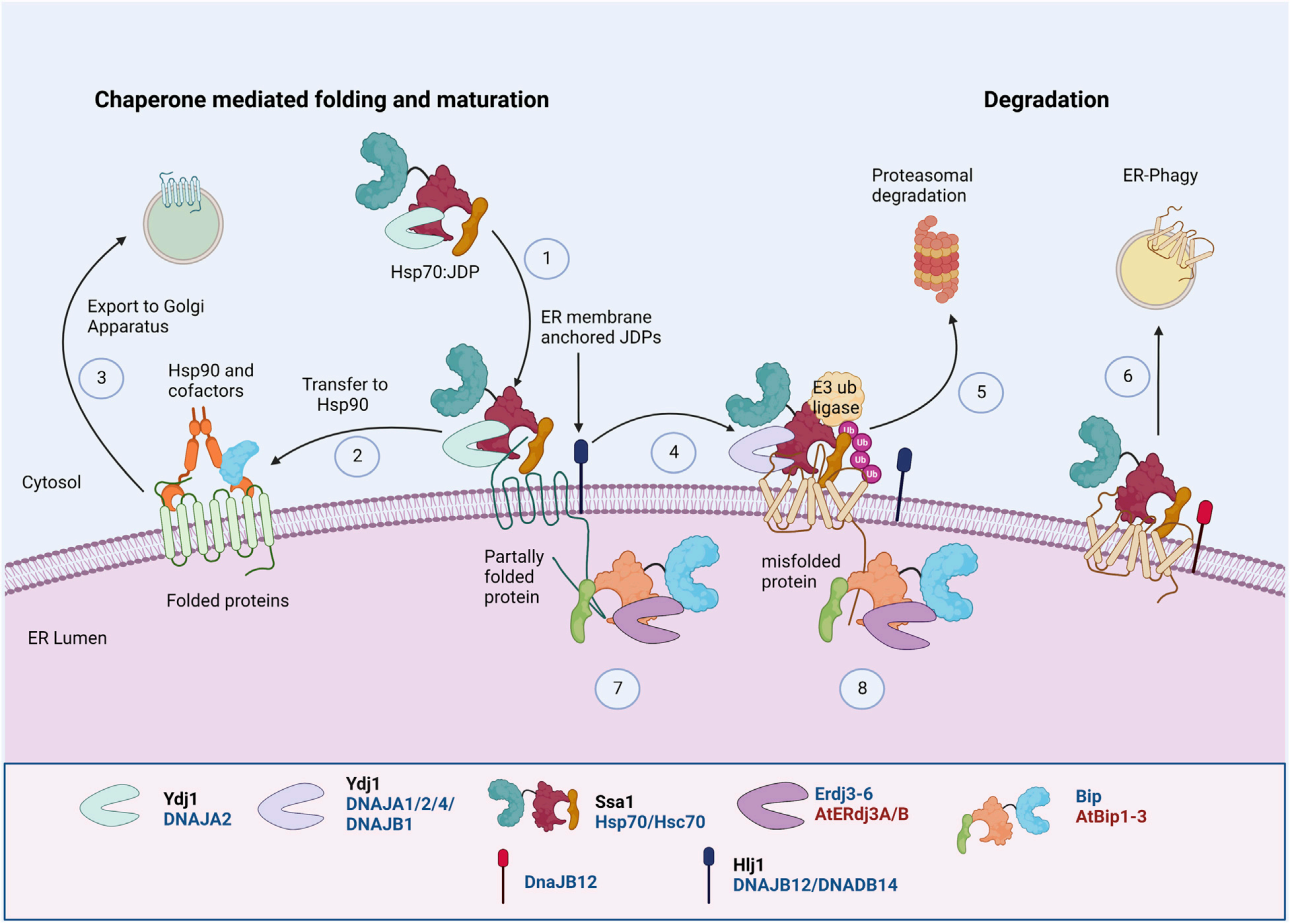
Protein Quality Control of PM proteins in ER membrane. PM proteins’ folding and maturation at ER involves both cytosolic and ER luminal Hsp70:JDP machineries. The cytosolic Hsp70:JDPs regulate the PQC by interacting with unfolded/partially folded proteins at the cytosolic domain^Y, H^ (1), mediating the interaction with Hsp90 for folding ^Y, H^ (2) and further trafficking to Golgi (3) (Youker et al., 2004; Donnelly et al., 2013; Li et al., 2017). Defect in proper folding and maturation, can re-direct them for ubiquitination^Y, H^ (4) and further proteasomal ^Y, H^ (5) or lysosomal degradation^H^ (6) (Nakatsukasa et al., 2008; Kim and Skach, 2012; He et al., 2021). Similarly, the luminal Hsp70:JDPs regulate the folding, maturation ^A, H^ (7) and degradation^A, H^ (8) of PM proteins by interacting at the luminal domains (Park and Seo, 2015; Ji et al., 2016). ^Y^ Literature available in Yeast; ^H^ Humans; ^A^ Arabidopsis. Names of Hsp70 and JDPs are denoted in different colors; Black for Yeast; Blue for Human.

Transmembrane proteins with an extended cytosolic domain are acted upon by cytosolic chaperones. Hsp70, JDP, and Hsp90 are involved in the early biogenesis of multiple PM transporters by interacting with their cytosolic domains (Kim and Skach, 2012; Donnelly et al., 2013) (Figure 3). They sequentially pass through three complexes with different co-chaperone compositions to achieve their active conformation. First, Hsp70:JDPs interact with the substrates to form the “early complex”. It prevents the aggregation and misfolding of substrates and also targets the misfolded proteins for degradation *via* ERAD (Box 3). The substrate is then transferred from Hsp70 to Hsp90 by HOP, forming an “intermediate complex”. Interaction with Hsp90 leads to the formation of a “folding complex” and favors substrate maturation and stabilization (Figure 3).

The early folding of mammalian chloride channel CFTR at the ER requires Hsp90, Hsp70:DNAJB1/Hdj1, and Hsc70:DNAJA1/Hdj2 (Meacham et al., 1999; Farinha et al., 2002; Farinha and Amaral, 2005). Similarly, in HEK293T cells, Hsp70 (HSPA1A) and Hsc70 (HSPA8), along with JDP, DNAJA1, regulate the early QC of thiazide-sensitive NaCl cotransporter (NCC), which further undergo Hsp90 mediated folding and maturation (Donnelly et al., 2013). Hsp70:JDPs also aid in the oligomerization and maturation of several membrane proteins (Li et al., 2017; Needham et al., 2019). Mammalian JDPs, DNAJB12 and DNAJB14, stabilize and promote tetrameric assembly of UNC-103, hERG, and Kv4.2 K^+^ channel subunits *in vitro* through an HSP70-independent mechanism. The oligomerization of DNAJB12 is important for its functions (Li et al., 2017). These observations reinforce the idea that cytosolic chaperones are equally important and work as surrogates to augment ER chaperones in regulating the PQC of PM proteins.

## Protein quality control in Golgi

After the PM proteins pass through the ER, they are packaged into vesicles and transported to the Golgi apparatus, which acts as the second QC checkpoint. Only a few misfolded proteins escape the ER PQC, and Golgi helps in 1) the retrotranslocation of defective proteins to the ER, 2) targeting of protein for proteasomal degradation, or 3) lysosomal degradation. Golgi is a central hub for protein sorting and modification of secretory and PM proteins. Some unassembled PM proteins, which escape the ER PQC, and reach the *cis*-Golgi, are retrieved back to the ER by “retention in ER sorting receptor 1” (Rer1), which mediates ER-retrieval (these proteins might undergo ERAD). Some mutant PM proteins further escape ER PQC and Rer1 retrieval and reach the Golgi. There, they undergo Golgi-PQC and ESCRT (endosomal sorting complex required for transport)-dependent vacuolar degradation. Apart from vacuolar degradation, some proteins also undergo proteasomal degradation by “endosome and Golgi associated degradation (EGAD)”. However, the mechanism of substrate recognition and the decision of ER retrieval vs degradation remains unclear (Rosquete et al., 2018; Hellerschmied et al., 2019; Benyair et al., 2022; Schwabl and Teis, 2022). Although likely, the involvement of an Hsp70:JDP in this process is not reported as yet.

## Folding and stability at the PM

PM proteins, passing through multiple subcellular organelles, finally reach their destination, the PM, where various extrinsic, as well as intrinsic factors regulate their function/stability. Moreover, PM is extremely dynamic, with continuous fusion and fission of membrane components. Further, the lipid and protein components of the PM are immensely variable depending on cellular physiology. PM proteins undergo conformational changes due to various perturbations and stimuli. These conformational changes are detected by the plasma membrane PQC (PMPQC) machinery and if required, the proteins are targeted for degradation (Nikko and Pelham, 2009; Zhao et al., 2013; Ghaddar et al., 2014). Little information is available on refolding of these proteins at the PM (Bagdany et al., 2017). Hsp70, along with Hsp90, are well known to regulate the conformational rearrangement and modulate the ligand binding activity of several cytosolic steroid receptors (Johnson and Craig, 2000; Pratt and Toft, 2003). It is likely that they regulate the stability and activity of PM proteins as well. A recent study underlined the role of Hsp70s in refolding and stabilizing the misfolded CFTR (Bagdany et al., 2017). Additionally, Hsp90 and Hsc70 stabilize the mature ΔF508CFTR in post-Golgi compartments. They reduce its protease susceptibility and shift the conformation towards native folding. Interestingly, active Hsc70 and DNAJA2, but not DNAJA1**,** are required for the conformational stabilization of the PM ΔF508CFTR at restrictive temperatures (Bagdany et al., 2017).

At the PM, the extracellular domains of the PM proteins are exposed and communicate with the outside environment. Defects in the extracellular domains are linked to various diseases (Accili et al., 1991; Amagai et al., 2015; Binder et al., 2018; Guo et al., 2019). Although the presence of proteases and chaperones, including Hsp70, BiP, and ERdj3, are reported in the extracellular space, the mechanism by which they regulate the proteins is yet to be established (De Maio, 2014; Wilson et al., 2022). Additionally, several Hsp70s are known to associate with lipids, interact with cell membranes, and even get inserted into lipid bilayers (Guidon and Hightower, 1986a; 1986b) reviewed in (De Maio and Hightower, 2021). Hsp70 (HSP1A) is known to interact with phosphatidylserine (PS) and insert into the PM during stress recovery to regulate a variety of cellular processes (Bilog et al., 2019). It has also been shown to induce pores in lipid membranes and insert into lipid bilayers to form stable ion conductors (Arispe and De Maio, 2000; Macazo and White, 2014). Only time will tell if these perform some canonical chaperone functions at the PM.

## Endocytosis

**FIGURE 4:**
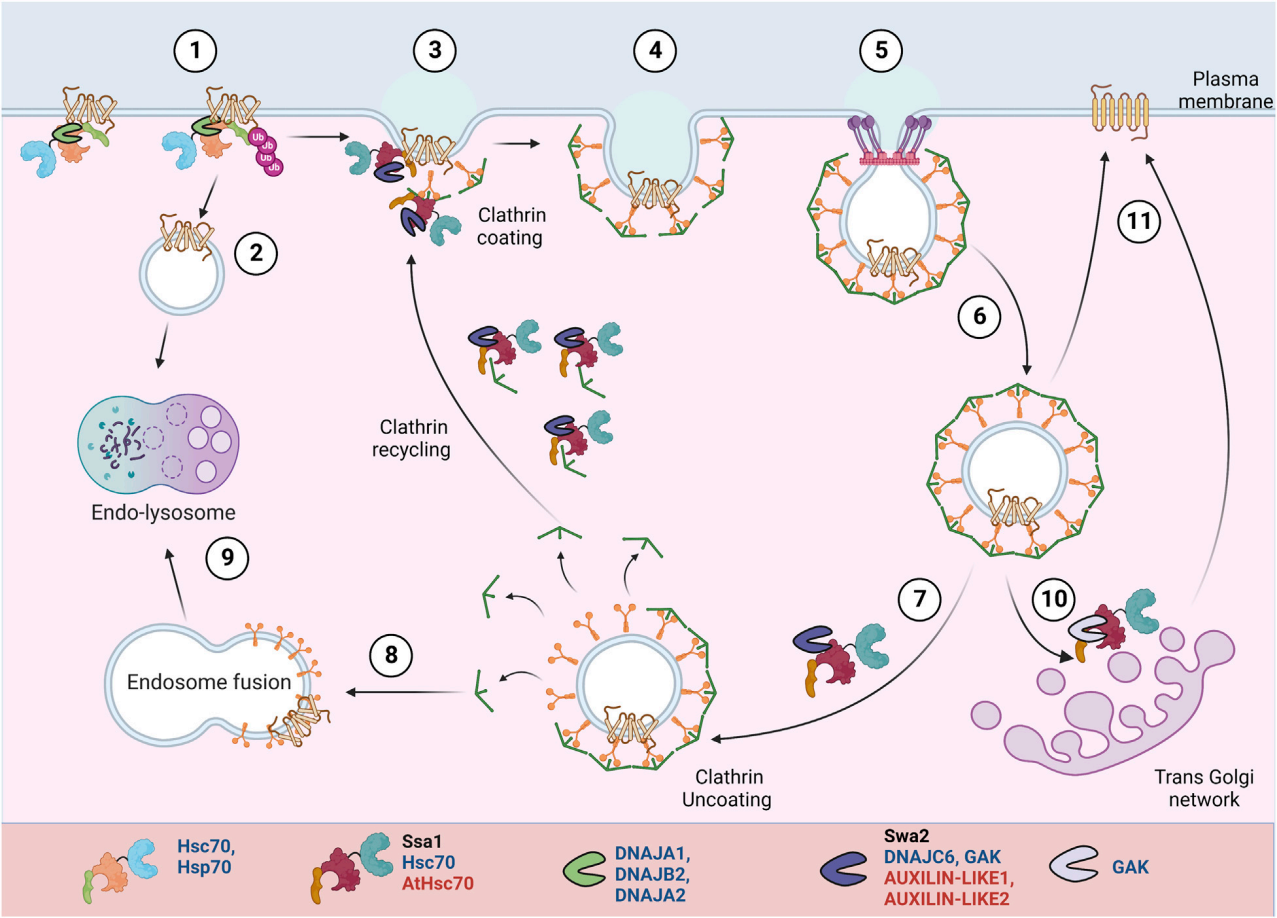
PM protein recycling/degradation by clathrin mediated endocytosis. In response to misfolding or stimuli mediated conformational changes, Hsp70:JDPs mediate PM proteins’ ubiquitination ^H^ (1) (Bagdany et al., 2017). The proteins undergo clathrin-independent (2) or clathrin-dependent (3-6) endocytosis. They can be targeted for lysosomal degradation (8-9) or recycled back to PM (10-11). A specific set of Hsp70:JDPs regulate clathrin-dependent endocytosis ^Y,H,A^, by mediating the membrane invagination, clathrin exchange, uncoating and recycling of clathrin to new vesicles (Greener et al., 2000; Xiao et al., 2006; Adamowski et al., 2018). ^Y^ Literature available in Yeast; ^H^ Humans; ^A^ Arabidopsis.Names of Hsp70 and JDPs are denoted in different colors; Black for Yeast; Blue for Human; Red for Arabidopsis.

PM proteins, once misfolded or no longer required, are ubiquitinated and removed from the PM. They can be further targeted for lysosomal degradation or can be recycled back to the PM after de-ubiquitination. PM proteins undergo endocytosis by several pathways, broadly categorized into clathrin-dependent or clathrin-independent mechanisms (Mayor and Pagano, 2007) (Figure 4). Clathrin-mediated endocytosis is the major and best-understood pathway for PM protein endocytosis in yeast, mammals, and plants (Lu et al., 2016; Kaksonen and Roux, 2018; Narasimhan et al., 2020). The process starts with the association of cytoskeletal factors and adaptor proteins near the substrate/cargo. It is followed by clathrin coat assembly and the release of the mature vesicle from the PM. Soon after, the vesicles are uncoated and fuse with endosomes. Some proteins in the early endosomes are deubiquitinated and are recycled back to the PM directly or *via* the trans-Golgi network. If not recycled, the ESCRT machinery targets them and forms MVBs, committing the proteins to lysosomal degradation.

Hsc70:Auxilin are essential for clathrin mediated endocytosis. During vesicle invagination, Hsc70:Auxilin are necessary for ATP-dependent clathrin exchange, thus mediating rearrangement of clathrin coat. At the end of each cycle, they moderate clathrin dissociation and possibly prevent their aggregation in the cytosol. Also, auxilin and Hsc70 may separately chaperone adaptor proteins, further helping to generate new clathrin coated pits (CCPs) (Jiang et al., 2000; Wu et al., 2001; Eisenberg and Greene, 2007; Sousa and Lafer, 2015).Yeast contains a single JDP, auxilin (Swa2), while in humans, Auxilin (Aux1/DNAJC6) and cyclin-G-associated kinase (GAK) Auxilin-2, regulate the clathrin dynamics (Xiao et al., 2006; Krantz et al., 2013; He et al., 2020). Auxilin (Aux1/DNAJC6) is nerve specific, while its homolog cyclin-G-associated kinase (GAK) or Auxilin-2 is ubiquitously expressed. Unlike auxilin, GAK is also enriched in the Golgi (Greener et al., 2000).

In yeast, normal cell development and endocytosis depend on the interaction of Swa2 (by J-domain and TPR-domain) with Hsp70, while Swa2-mediated clathrin binding is expendable (Gall et al., 2000; Xiao et al., 2006; Krantz et al., 2013; Tuo et al., 2013). In HeLa cells, transient GAK knockdown affected the transferrin uptake in a J-domain-dependent manner. The lack of J-domain did not affect its interaction with clathrin or other endocytic factors but disabled GAK’s ability to uncoat clathrin (Zhang et al., 2005). Stable depletion of GAK inhibits the uptake of epidermal growth factor (EGF), resulting in changes in downstream EGFR signaling and a 50-fold increase in the expression levels of EGF receptor (EGFR) (Zhang et al., 2004). Depletion of GAK reduces the trans-Golgi-associated perinuclear clathrin and PM CCPs. Besides clathrin uncoating, the depletion of GAK also partially inhibited trafficking between the trans-Golgi network (TGN) and lysosomes. It alters the Golgi morphology, probably by affecting the clathrin adaptors (Zhang et al., 2005, 2004; Lee et al., 2005). In plants as well, the role of Hsp70:JDP in uncoating of CCV (Clathrin coated vesicles) is reported. AUXILIN-LIKE1 stimulates vesicle uncoating in the presence of HSC70 and interacts with SH3P1 and clathrin *in vitro* (Lam et al., 2001). *In planta* studies showed that overexpression of both AUXILIN-LIKE1 and 2 leads to inhibition of endocytosis, most likely by preventing clathrin recruitment to endocytic pits. They result in an arrest in seedling growth and development. However, auxilin-like1/2 loss-of-function mutant does not result in endocytic or developmental defects, suggesting a possible redundant role of AUXILIN-LIKE 3-7 proteins (Adamowski et al., 2018).

## Degradation of PM proteins

Genetic mutations, biosynthetic errors or cellular stress can cause misfolding and aggregation of proteins. Clearing of non-functional or cytotoxic PM proteins by different cellular degradation machineries is essential for maintaining cellular homeostasis. Luckily, for PM proteins, such PQC checkpoints operate right from their synthesis in the cytosol, their movement through the endomembrane system to their final destination in the PM. Hsp70s and JDPs have a major role in the solubilization of protein aggregates and the degradation of unfolded proteins (Rosenzweig et al., 2019). Interaction of Hsp70:JDPs with the degradation-competent clients may act as a signal for the recruitment of E3 ubiquitin ligases, thereby targeting proteins for protein degradation (Buchberger et al., 2010). Additionally, Hsp70:JDPs interact with the substrate to avoid protein aggregation or keep it in a competent state for ubiquitination and further targeting to proteasomal degradation (Vembar and Brodsky, 2008; Shiber et al., 2013). Below we discuss examples where Hsp70:JDPs systems target specific PM proteins for degradation, if deemed unfit.

### Failure to enter the ER

Failure of PM proteins to enter the ER results in ubiquitination, either co- or post-translationally, followed by their proteasomal degradation (Wang et al., 2013; Tian et al., 2021). Yeast cytosolic Hsp70:JDP (Ydj1) mediate proteasomal degradation of import-incompetent proteins (Park et al., 2007). Similarly, in human MCF-7 cell lines, Hsp70 and DNAJB1 mediate rapid nascent polypeptide degradation during heat stress (Tian et al., 2021). The mammalian Hsp70 and DNAJA1 interact and facilitate the degradation of the PM protein, Apolipoprotein B (ApoB) (Kumari et al., 2022). In lipid-deficient conditions, ApoB does not enter the ER and is deposited in the cytoplasm, where it is targeted for degradation by Hsp70, Hsp90, Hsp110, and the ER membrane-associated JDP, p58IPK (Zhou et al., 1998; Kumari and Brodsky, 2021).

### Defects in folding and maturation in the ER

The ER harbors a very stringent PQC network where the quality and quantity of proteins are tightly regulated. However, defects in protein folding or maturation often lead to its degradation by ER-associated degradation (ERAD) (Box 3) or selective ER autophagy (Anelli and Sitia, 2008; Chino and Mizushima, 2020). Hsp70:JDPs regulate the folding, maturation as well as degradation of PM proteins both in the cytosol and the ER lumen (Figure 3). A vast portion of PM proteins are transmembrane proteins and are acted upon by both cytosolic and luminal PQC components. The Hsp70:JDPs interact with the misfolded proteins to mediate their ubiquitination and maintain them in a retro-translocation competent state (Silberstein et al., 1998; Gillece et al., 1999; Nishikawa et al., 2001; Vembar and Brodsky, 2008; Xie and Ng, 2010; Shiber et al., 2013; Pobre et al., 2019). Upon ubiquitination, the ERAD substrates are retrotranslocated to the cytosol by the AAA-ATPase, Cdc48, and targeted for proteasomal degradation (Wolf and Stolz, 2012). In yeast, Hsp70 (Ssa1), Hsp42, and Hsp100 interact with the substrates to prevent aggregation (Shiber et al., 2013; Doonan et al., 2019).

#### Role of luminal JDPs

In budding yeast, luminal JDP, Scj1, interacts with E3 ubiquitin ligase, Hrd1, for the degradation of soluble proteins. However, the luminal Hsp70:JDPs have little effect on the degradation of membrane proteins (Plemper and Wolf, 1999; Nishikawa et al., 2001; Mehnert et al., 2015). In higher eukaryotes, the role of ER luminal JDPs in regulating PM proteins is more evident. In human m17 neuroblastoma cells, Hsp70, BiP, targets the GPI-anchored mutant prion protein, PrP Q217R, for proteasomal degradation (Jin et al., 2000). A study in mice showed that BiP regulates the degradation of pre-B cell receptors (pre-BCR), thus regulating pre-BCR signaling (Ji et al., 2016). Similarly, in HEK293 cells, BiP, along with ERdj4 and ERdj5, regulate the ERAD of misfolded surfactant protein C (SP-C) proprotein associated with human interstitial lung disease (Dong et al., 2008). Erdj4 (DNAJB9) also physically interacts and regulates the expression of cystic fibrosis transmembrane conductance regulator (CFTR) at the cell surface (Huang et al., 2019).

Interestingly, luminal JDPs can also regulate the degradation of substrates, independent of Hsp70. One of the ERAD substrates, the mammalian Epithelial sodium channel (ENaC), is composed of α-, β-, and γ-subunits. Nearly 75% of each subunit resides in the ER lumen and membrane, and ∼25% resides in the cytoplasm. In the kidney, the β- and γ-subunits are constitutively expressed at much higher levels than the α-subunit. In the absence of the α-subunit, the β- and γ-subunits undergo degradation. Jem1, Scj1 (in yeast), and Erdj3, Erdj4 (in Xenopus oocyte) can function independently of luminal Hsp70 to facilitate ENaC degradation (Buck et al., 2010).

#### Role of cytosolic JDPs

Often the large, multimeric PM proteins harbor an extended cytosolic domain. Cytosolic Hsp70:JDPs often interact with these domains to regulate their maturation or degradation. In budding yeast, two JDPs, namely Hlj1 and Ydj1, regulate the degradation of various misfolded PM proteins, including mammalian protein CFTR, ABC transporter Ste6* and Pma1-D378S mutant (Zhang et al., 2001; Huyer et al., 2004; Youker et al., 2004; Han et al., 2007; Nakatsukasa et al., 2008). Both Hlj1 and Ydj1 associate with the ER and act in an Ssa1-dependent manner. Interestingly, the majorly cytosolic JDP, Sis1, also regulates the ubiquitination of a subset of ERAD substrates; however, its Hsp70 co-chaperone activity seems to be dispensable for the function (Shiber et al., 2013). It is suggested that depending on the extent of misfolding, the role of Ssa1 might be dispensable for substrate ubiquitination (Shiber et al., 2013). This is not particularly surprising, as many JDPs carry out chaperone functions that do not require their J-domain (Ajit Tamadaddi and Sahi, 2016). JDPs, Cwc23, and Jid1, are also reported to affect the ERAD of certain substrates in unknown ways (Taxis et al., 2003). However, later studies found Jid1 to be localized to mitochondria and Cwc23 to have nuclear functions (Bursać and Lithgow, 2009; Sahi et al., 2010). Hence, the observed effects might not be direct.

In mammalian systems, CFTR is one of the most extensively studied PM proteins. The most prevalent CFTR mutation, ∆F508, is found in ∼90% of cystic fibrosis (CF) patients, where it impairs CFTR folding, inhibits channel gating, and decreases PM stability. Cytosolic (DNAJB1/Hdj1, DNAJA1/Hdj2) and ER membrane JDPs (DNAJB12, DNAJB14) work with cytosolic and luminal Hsp70s and Hsc70, respectively, to oversee the ER-PQC of CFTR (Meacham et al., 1999; Choo-Kang and Zeitlin, 2001; Farinha et al., 2002; Grove et al., 2011; Sopha et al., 2012; Li et al., 2017). *In vitro* studies suggest that the co-translational interaction of Hsc70 makes CFTR less susceptible to degradation, while post-translational interaction favors degradation (Matsumura et al., 2011). Co-overexpression of Hsp70 and DNAJB1 in Baby hamster kidney (BHK) cells stabilized the immature form of wildtype CFTR but not mutant ∆F508 CFTR (Farinha et al., 2002). In HeLa cells, DNAJA1 and Hsc70 interact with the cytosolic domain of misfolded (∆F508 CFTR) as well as wild-type CFTR. While the misfolded protein is targeted for degradation, chaperones interact with the wild-type CFTR only until it attains its tertiary structure. The levels of complex formation between ∆F508 CFTR and DNAJA1/Hdj2-Hsp70 are approximately 2-fold higher than those with wild-type CFTR (Meacham et al., 1999; Grove et al., 2011), suggesting additional chaperone assistance required by the mutant CFTR as compared to the wild-type protein. Another mammalian channel protein, ENaC, is differentially regulated by Hsc70 and Hsp70. While overexpression of Hsc70 favors the degradation, Hsp70 stabilizes the protein, thus modulating its trafficking and surface expression (Chanoux et al., 2013; Kang and Jeon, 2021). The Hsp70:DNAJA1 regulate the degradation of CFTR and other proteins *via* the CHIP E3 Ub ligase mediated ubiquitination. Similarly, NaCl cotransporter (NCC), whose loss of function results in Gitelman syndrome, is regulated by cytosolic Hsp70-JDPs. HEK293T cells expressing Gitelman mutants of NCC showed enhanced association with Hsp70 and Hsp40 as compared to the wild-type protein leading to higher CHIP-mediated degradation (Donnelly et al., 2013).

The ER membrane anchored JDPs, DNAJB12 and DNAJB14, also regulate the QC of PM proteins at their cytosolic domain. Modest elevation of DNAJB12 (but not its J-domain mutant, DNAJB12-QPD) decreased the accumulation of both wild-type and mutant (ΔF508) CFTR by increasing its association with Hsc70 and the ubiquitin E3 ligase, RMA1. Depletion of DNAJB12 increased CFTR folding efficiency up to three-fold and permitted a pool of ΔF508 CFTR to fold and escape the ER (Grove et al., 2011). It is suggested that DNAJB12/14 enhance the protein degradation in an Hsp70-dependent manner to regulate the maturation of proteins in cells. DNAJB12/14, although involved in ERAD of CFTR and gonadotropin-releasing hormone receptor mutant, S168R-GnRHR (Houck et al., 2014), they do not participate in ERAD of misfolded hERG (K^+^ channel) proteins (Li et al., 2017). Rather, DNAJA1, DNAJA2, and DNAJA4 overexpression in HEK-293 and HeLa cells inhibited the hERG maturation and trafficking in an Hsc70-dependent manner (Walker et al., 2010). It suggests the specificity of JDPs in handling different PM proteins.

Besides the ERAD pathways, multiple PM proteins are also degraded by selective ER-autophagy (Figure 3). Often, large misfolded membrane proteins can accumulate in complex tertiary structures, which are difficult to unfold and retrotranslocate. These ERAD-resistant clients are degraded by ER autophagy, for e.g., misfolded gonadotropin-releasing hormone receptor (GnRHR) E90K-GnRHR and N1303K-CFTR (Houck et al., 2014; He et al., 2021). Intuitively, inefficient ERAD also leads to the autophagy of some substrates. Although the mechanism is not completely understood, Hsp70 and DNAJB12 have been shown to interact with the autophagy machinery in the presence of specific complex substrates. Depletion of DNAJB12 inhibits the lysosomal targeting of N1303K-CFTR in HEK293 cells. Also, the overexpression of the DNAJB12-QPD mutant (J-domain mutant) prevented ER phagy. Hence, it acts with the partner Hsp70 to regulate ER phagy of complex substrates (He et al., 2021). However, at this point, the involvement of other Hsp70:JDP machinery in ER autophagy cannot be ruled out.

### Removal of proteins from PM

PM Proteins destined to be degraded are recognized at their exposed cytosolic domain. Cytosolic adaptors (in yeast) or chaperones (in mammals) interact with the substrates, recognize the conformational changes, and recruit the E3 ubiquitin ligases (Babst, 2014; MacGurn, 2014). Ubiquitination of proteins ensures their endocytosis and fusion with early endosomes. Further, they can be deubiquitinated and recycled back to the PM directly or through Golgi; else, they are targeted for MVB sorting and undergo lysosomal degradation (Figure 4).

In yeast, the major components of the PMPQC pathway include the ART-Rsp5 system, which recognizes, ubiquitinates, and targets proteins for degradation. Although the turnover of multiple PM proteins is regulated by E3-ubiquitin ligase, Rsp5, the degradation of misfolded PM proteins in yeast is poorly understood. To this end, it is even proposed that in budding yeast, PM proteins with an exposed misfolded cytosolic domain might be escaping the PMPQC network and may not be as efficiently degraded (Lewis and Pelham, 2009). The multi-functional JDP Ydj1, although it operates with Rsp5 for cytosolic heat-denatured substrates, it is not involved in the QC of PM proteins (Fang et al., 2014). It is still an open question if another JDP operates with Rsp5 for the degradation of PM proteins. Nevertheless, Caj1**,** a cytosolic JDP in budding yeast, negatively regulates the Rsp5-mediated degradation of tryptophan transporters Tat1 and Tat2 (Welsch et al., 2003; Hernández-López et al., 2011; Dobriyal et al., 2020). Interestingly, Caj1 also associates with different lipids, reportedly phosphatidic acid and phosphatidylinositol-5-phosphate. Besides being majorly cytosolic, Caj1 is partially localized to the PM as well (Dobriyal et al., 2020; Herianto et al., 2021; Zhang et al., 2021). Overexpression of Caj1 (but not Caj1 H34Q mutant) stabilized tryptophan permeases Tat1 and Tat2, suggesting a possible role of Hsp70:JDP machinery in regulating the stability/turnover of PM proteins in budding yeast.

In mammals, a more elaborate PMPQC network has been deciphered that regulates the turnover of misfolded proteins. Several molecular chaperones and co-chaperones recognize and decide the fate of the misfolded PM proteins (Okiyoneda et al., 2011). Similar to the chaperone-mediated substrate handling at the ER, defective PM protein degradation is regulated by JDP-Hsp70-Hsp90s. However, Hsp70/HSPA1A, Hsc70/HSPA8, DNAJA1, and DNAJB2 are among the major components of PMPQC, deciding the fate of misfolded proteins (Okiyoneda et al., 2011). They help in the ubiquitination of proteins by the recruitment of the E3 ubiquitin ligase, CHIP (C-terminal Hsp70 interacting protein) (Connell et al., 2001). Hsp70, through its C-terminal EEVD motif, directly interacts with the N-terminal TPR domain of CHIP. The interaction stalls protein folding and concomitantly facilitates the ubiquitination of Hsp70-bound substrates. In this process, the JDP stimulates the chaperone activity of Hsp70 and CHIP and increases client ubiquitination (Rosser et al., 2007; Zhang et al., 2015).

Several misfolded PM proteins undergo Hsp/c70-JDP-CHIP mediated degradation. For example, the mature, glycosylated ΔF508CFTR interacts with Hsp70 and DNAJB2/Hdj1 under restrictive conditions in post-Golgi compartments in BHK-21 cells (Bagdany et al., 2017). Si-RNA mediated downregulation of Hsc70 (HSPA8), DNAJB2 (Hdj1), DNAJA1 (Hdj2), and DNAJC7 (TPR2) caused the accumulation of mutant ΔF508CFTR at the PM of HeLa cell lines (Okiyoneda et al., 2010). Similarly, JDP, Hsp70, Hsc70, and Hsp90, along with CHIP, are known to interact with the unfolded protein CD4T-λC at the PM but not with the native protein to facilitate ubiquitination and degradation (Apaja et al., 2010). CHIP-mediated PQC was also found for dopamine D4.4 receptor (DRD4) and vasopressin V2 receptor (V2R) mutants, which escape the secretory pathway PQC, and undergo rapid degradation at the PM (Apaja et al., 2010). In yet another case, defects in the turnover of the hERG K^+^ channel, which leads to long QT syndrome 2 (LQT2), is also regulated by Hsp70-CHIP. Disruption of Hsp70’s interaction with CHIP prevented substrate-based rapid endocytosis of both wild-type as well as unstable mutant hERG from the PM (Apaja et al., 2013). In the human collecting duct (mpkDCT and mpkCCD cells), CHIP-Hsc70 directly interact and regulate the degradation of water channel aquaporin (AQP2), thus maintaining the urine osmolarity and renal water handling (Lu et al., 2007; Wu et al., 2018). A JDP co-chaperoning Hsp70 in these processes is yet to be identified.

## Conclusion and perspectives

PM proteins are essential for life. During their lifetime, they pass through a series of rigorous PQC checkpoints that ensures their proper folding, stability, functionality, and finally, their abundance in the cell. Hsp70:JDP networks in the cytosol, the ER, as well as at the PM are emerging as the key players that assist in the trafficking, biogenesis, maturation as well as degradation of PM proteins. As they navigate inside the cell, PM proteins are relayed through many different JDPs. And depending on the type of client, JDPs may have pro-folding or pro-degradation functions. Understanding how Hsp70:JDP machinery manages to perform this task is the Achilles heel of this field. Interestingly, multiple JDPs sequentially operate with a single pool of Hsp70 to perform rather unique PQC functions on a single PM protein, suggesting highly intricate and regulated interactions between JDPs with client proteins and with Hsp70. Finally, the discovery of lipid interacting and PM-associated Hsp70s and JDPs is bound to bring about a paradigm shift in our understanding of how these versatile chaperones cater to the needs of PM proteins at the plasma membrane.

BOX 1 **Hsp70: J-domain protein (JDP) machinery**

Hsp70s, the 70 KDa molecular chaperones, are the key players in cellular proteostasis. They work in collaboration with two other components of the chaperone network, i.e., Hsp40s (also called J-domain proteins or JDPs) and Nucleotide exchange factors (NEFs) (Mayer and Bukau, 2005; Young, 2010; Rosenzweig et al., 2019). Hsp70s have a C-terminal Substrate Binding Domain (SBD), through which they bind to the hydrophobic regions of their client proteins, and an N-terminal Nucleotide Binding Domain (NBD), which binds to ATP and hydrolyzes it to ADP. The ATPase activity of Hsp70 is central to all its chaperone functions as this allosterically regulates its client-binding properties. However, the weak intrinsic ATPase activity of Hsp70s is not sufficient for its chaperone functions. JDPs, the obligate co-chaperones of Hsp70s, have a conserved J-domain with an invariant HPD motif responsible for stimulating the ATPase activity of Hsp70s, thereby potentiating its chaperone functions. The immense versatility of Hsp70 stems from their ability to operate with multiple JDPs in any given compartment, where, besides stimulating the ATPase activity of Hsp70s, they also determine the multi-valency of Hsp70 chaperones (Qiu et al., 2006; Craig and Marszalek, 2017; Rosenzweig et al., 2019; Liu et al., 2020). Furthermore, JDPs can also specify Hsp70s function by tethering Hsp70s to a specific subcellular localization (Kampinga and Craig, 2010). JDPs are highly diverse in their structure, function as well as their cellular abundance. Different cellular compartments harbor multiple JDPs performing general or highly specialized functions, ranging from folding, transport, and degradation of proteins, to mediating protein-protein interactions and assembly or disassembly of protein complexes. While JDPs form obligate partnerships with the Hsp70 chaperone, there is increasing evidence that some JDPs may have functions outside this association as well.

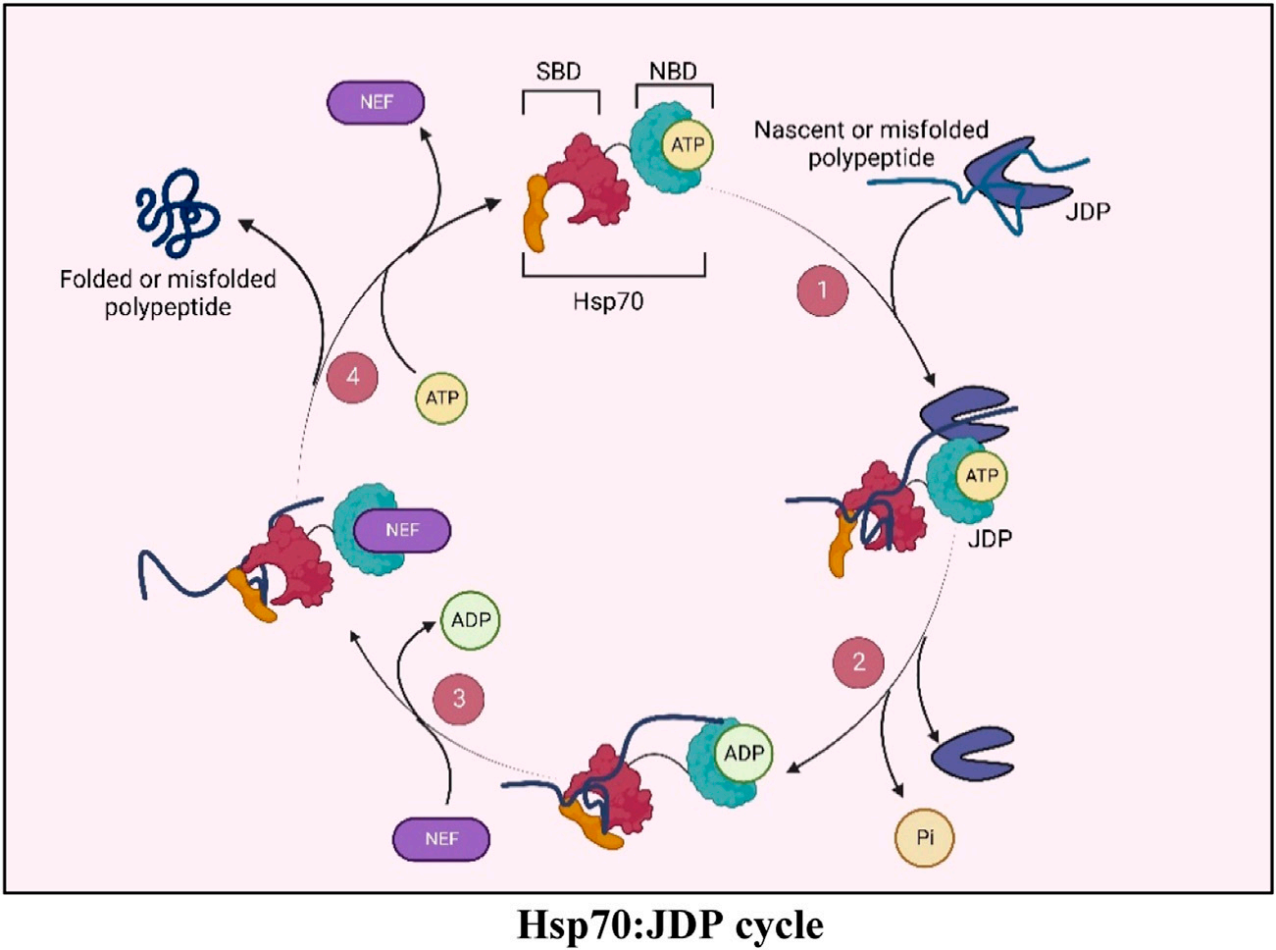

BOX 2 **UPR**

Unfolded protein response (UPR) is an evolutionarily conserved ER (Endoplasmic reticulum) stress response pathway to regulate the cell’s optimal capacity for protein synthesis and maturation. It is primarily induced by the accumulation of aberrant proteins in the ER due to various environmental stimuli, activation of other cellular stress response pathways, or lack of efficient protein folding machinery in the ER. UPR signaling pathways communicate the ER-stress to the gene expression machinery in the nucleus and cytosol, and regulate cellular transcription and translation. UPR restores ER homeostasis by 1) expanding the ER membrane and increasing ER abundance to meet protein folding demands, 2) positively regulating protein folding machinery, 3) activating the ERAD (ER-associated degradation) pathway etc. Further, under aggravated and long-term ER stress, UPR signaling may lead to apoptotic cell death (Walter and Ron, 2011; Hetz et al., 2020; Read and Schröder, 2021).

BOX 3 **ERAD**

The Endoplasmic reticulum-associated degradation (ERAD) is an extensive PQC network that oversees the regulated degradation of ER proteins. ER is the primary site for the folding and maturation of endomembrane, secretory and PM proteins, and failure to reach mature confirmation targets these proteins for degradation. ERAD pathway mediates the removal of unfolded, misfolded or unwanted proteins from the ER (Vembar and Brodsky, 2008). ERAD has three major branches based on the site of protein misfolding. Proteins with a lesion on the luminal, transmembrane, and cytosolic domain are targeted by ERAD-L, ERAD-M, and ERAD-C pathways, respectively. Different classes of molecular chaperones, ubiquitination machinery, and other PQC factors regulate these pathways. It involves recognition of misfolded substrates by chaperones, its ubiquitination by E2 ub conjugating enzymes and E3 ub ligases, extraction out of ER by retrotranslocons, and finally, targeting to the proteasome for degradation (Kumari and Brodsky, 2021).
